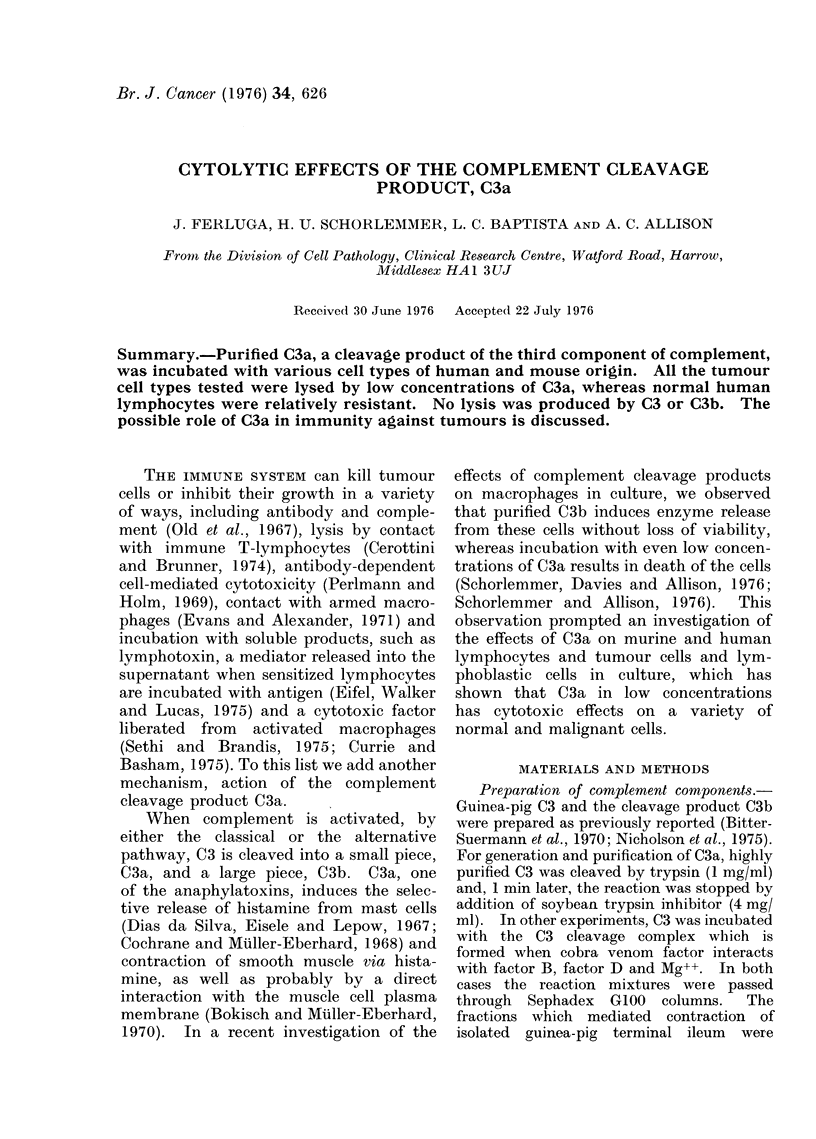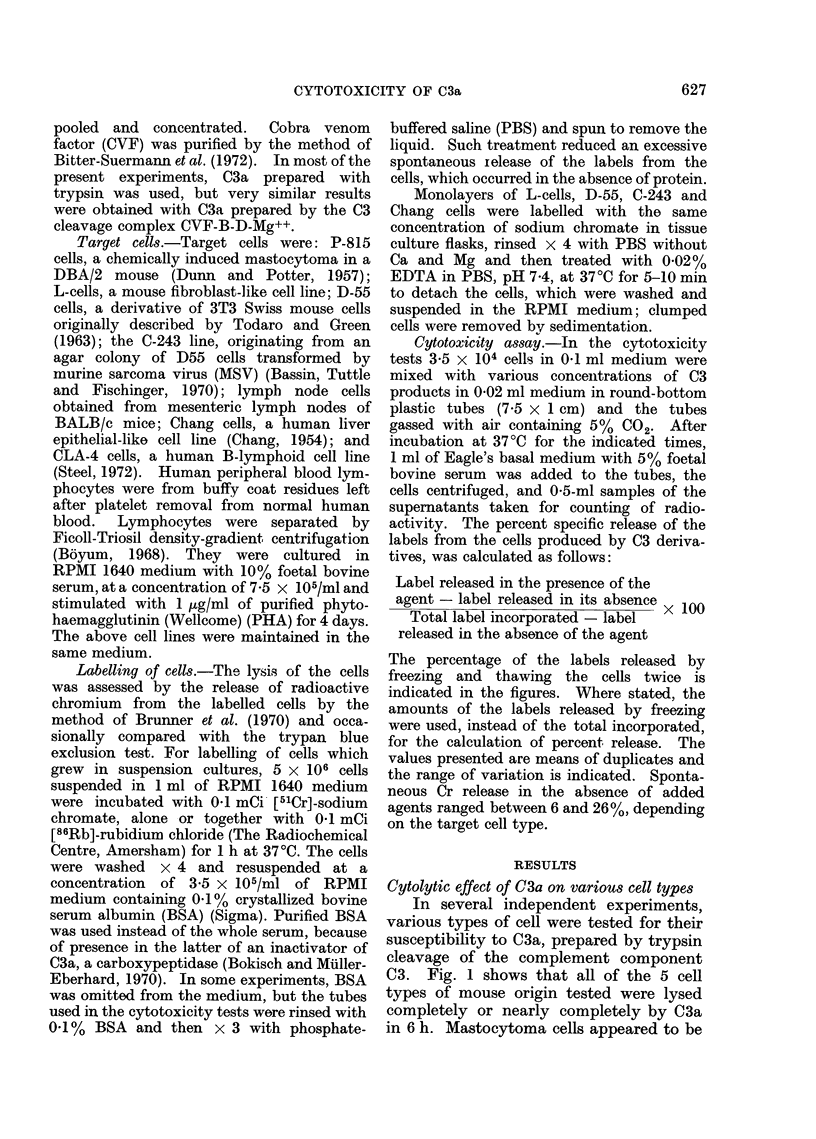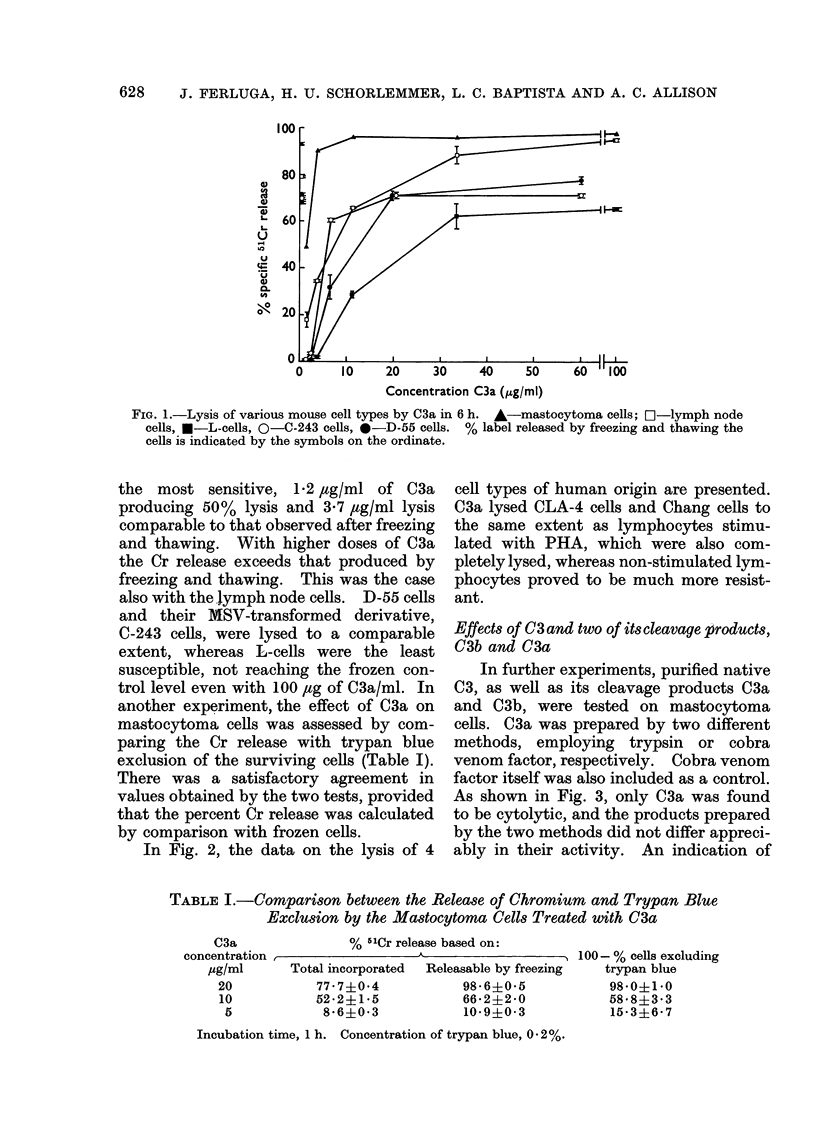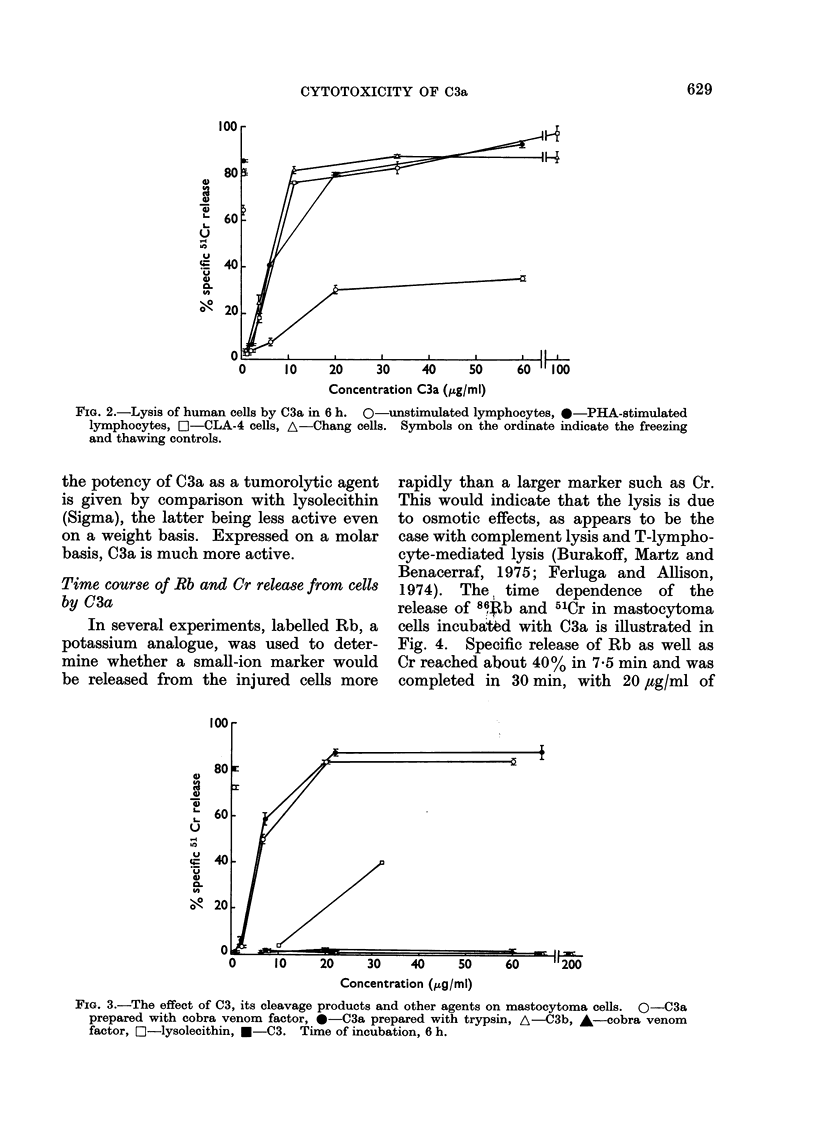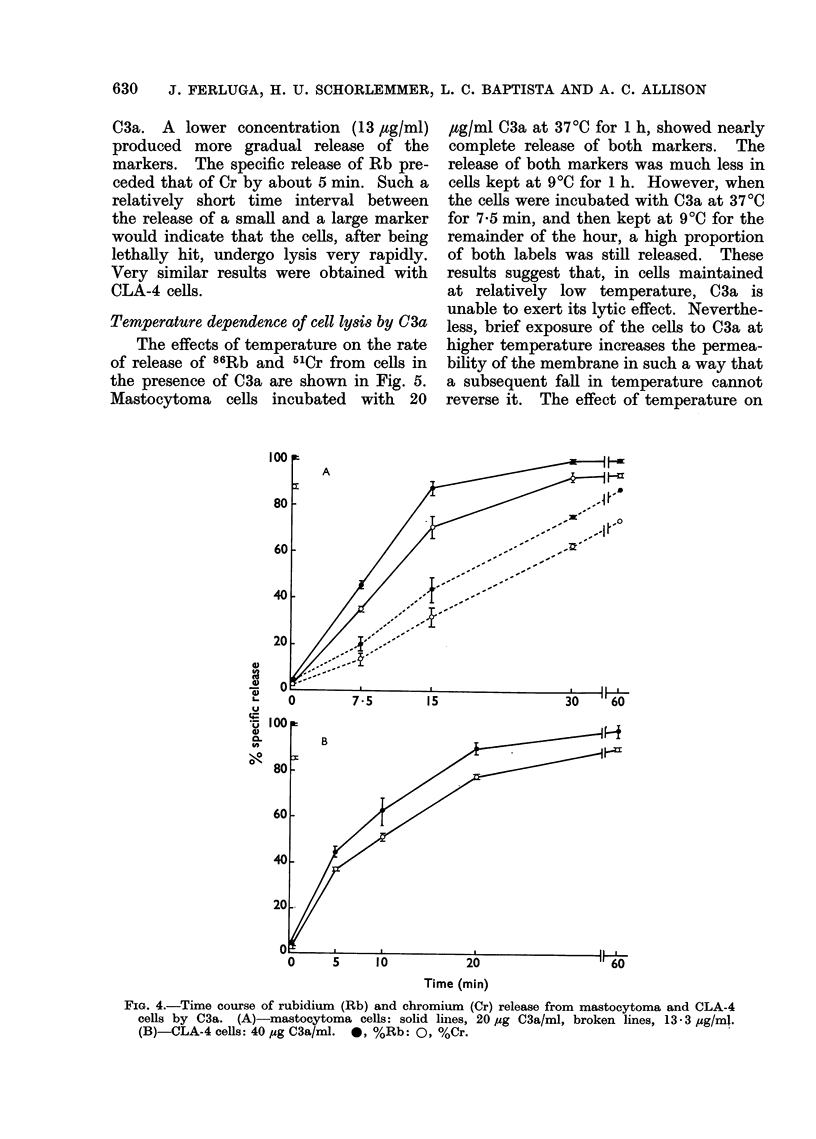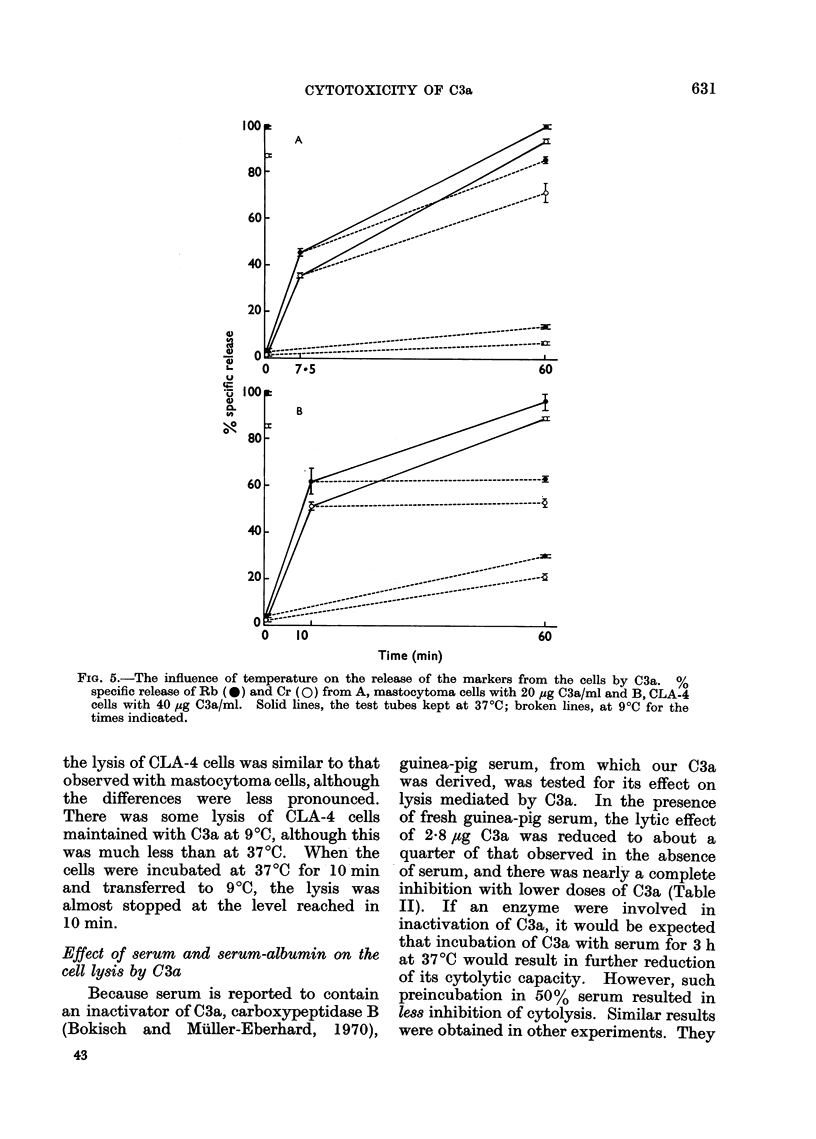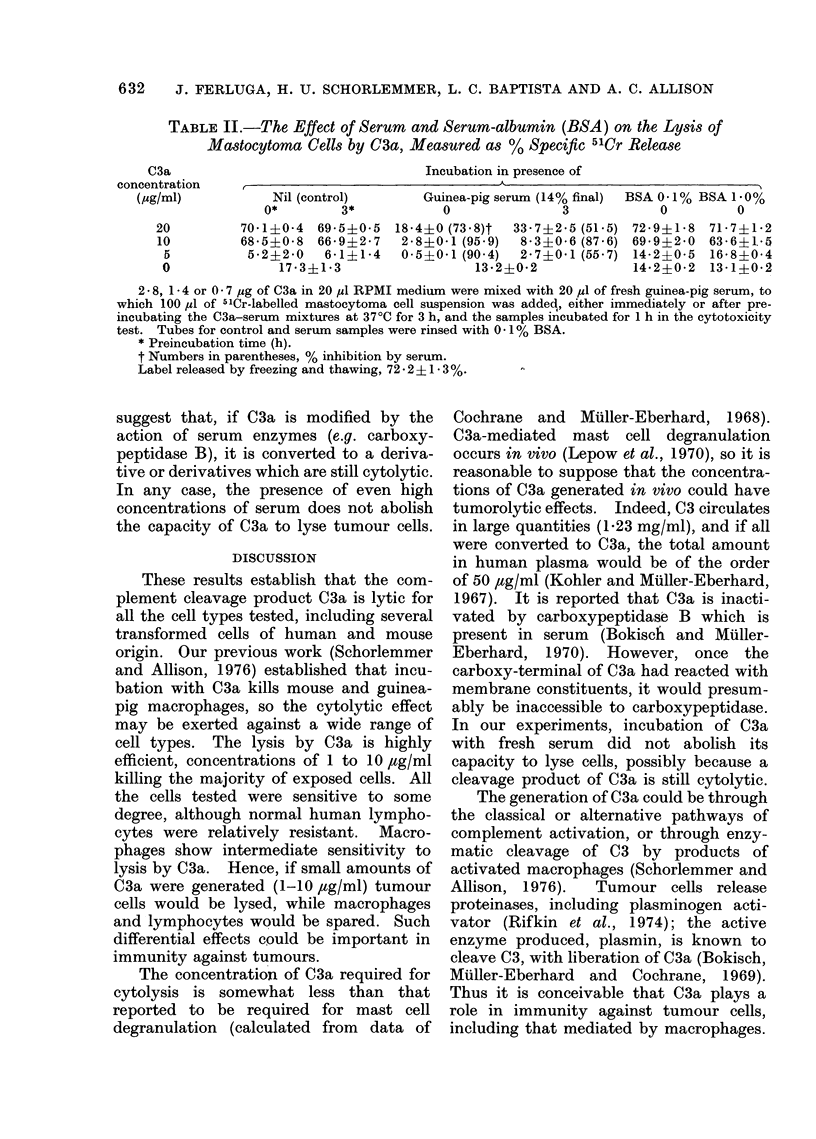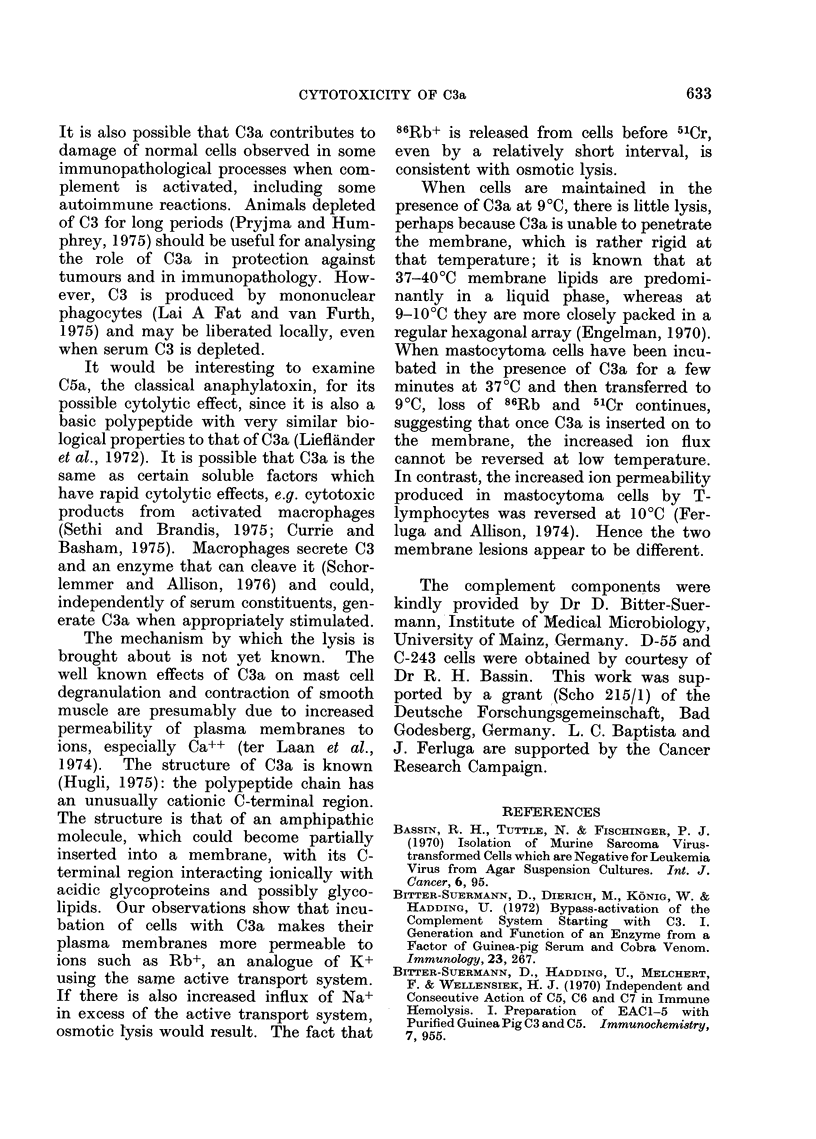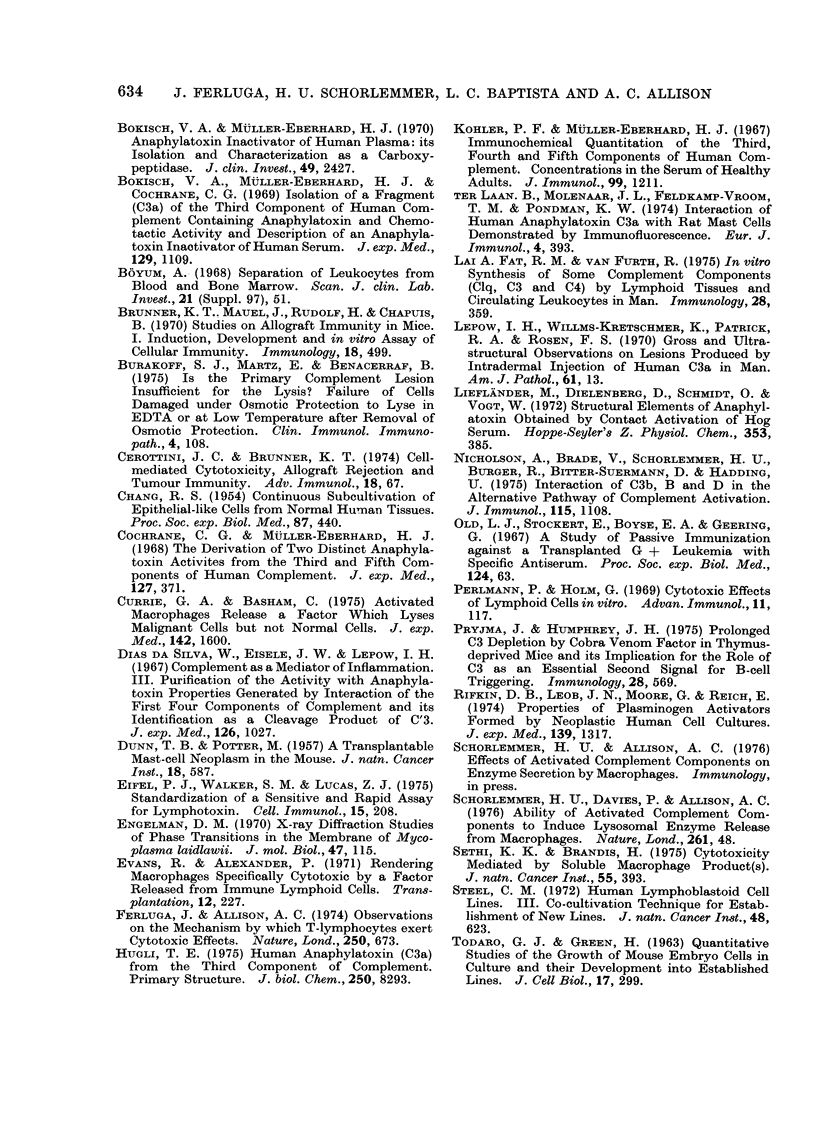# Cytolytic effects of the complement cleavage product, C3a.

**DOI:** 10.1038/bjc.1976.223

**Published:** 1976-12

**Authors:** J. Ferluga, H. U. Schorlemmer, L. C. Baptista, A. C. Allison

## Abstract

Purified C3a, a cleavage product of the third component of complement,was incubated with various cell types of human and mouse origin. All the tumour cell types tested were lysed by low concentrations of C3a, whereas normal human lymphocytes were relatively resistant. No lysis was produced by C3 or C3b. The possible role of C3a in immunity against tumours is discussed.


Br. J. Cancer (1976) 34, 626

### CYTOLYTIC EFFECTS OF THE COMPLEMENT CLEAVAGE PRODUCT, C3a

J. FERLUGA, H. U. SCHORLEMMER, L. C. BAPTISTA AND A. C. ALLISON

*From the Division of Cell Pathology, Clinical Research Centre, Watford Road, Harrow, Middlesex HA1 3UJ*

Received 30 June 1976 Accepted 22 July 1976

**Summary.—Purified C3a, a cleavage product of the third component of complement, was incubated with various cell types of human and mouse origin. All the tumour cell types tested were lysed by low concentrations of C3a, whereas normal human lymphocytes were relatively resistant. No lysis was produced by C3 or C3b. The possible role of C3a in immunity against tumours is discussed.**

THE IMMUNE SYSTEM can kill tumour cells or inhibit their growth in a variety of ways, including antibody and complement (Old *et al.*, 1967), lysis by contact with immune T-lymphocytes (Cerottini and Brunner, 1974), antibody-dependent cell-mediated cytotoxicity (Perlmann and Holm, 1969), contact with armed macrophages (Evans and Alexander, 1971) and incubation with soluble products, such as lymphotoxin, a mediator released into the supernatant when sensitized lymphocytes are incubated with antigen (Eifel, Walker and Lucas, 1975) and a cytotoxic factor liberated from activated macrophages (Sethi and Brandis, 1975; Currie and Basham, 1975). To this list we add another mechanism, action of the complement cleavage product C3a.

When complement is activated, by either the classical or the alternative pathway, C3 is cleaved into a small piece, C3a, and a large piece, C3b. C3a, one of the anaphylatoxins, induces the selective release of histamine from mast cells (Dias da Silva, Eisele and Lepow, 1967; Cochrane and Müller-Eberhard, 1968) and contraction of smooth muscle *via* histamine, as well as probably by a direct interaction with the muscle cell plasma membrane (Bokisch and Müller-Eberhard, 1970). In a recent investigation of the effects of complement cleavage products on macrophages in culture, we observed that purified C3b induces enzyme release from these cells without loss of viability, whereas incubation with even low concentrations of C3a results in death of the cells (Schorlemmer, Davies and Allison, 1976; Schorlemmer and Allison, 1976). This observation prompted an investigation of the effects of C3a on murine and human lymphocytes and tumour cells and lymphoblastic cells in culture, which has shown that C3a in low concentrations has cytotoxic effects on a variety of normal and malignant cells.

### MATERIALS AND METHODS

*Preparation of complement components.*—Guinea-pig C3 and the cleavage product C3b were prepared as previously reported (Bitter-Suermann *et al.*, 1970; Nicholson *et al.*, 1975). For generation and purification of C3a, highly purified C3 was cleaved by trypsin (1 mg/ml) and, 1 min later, the reaction was stopped by addition of soybean trypsin inhibitor (4 mg/ml). In other experiments, C3 was incubated with the C3 cleavage complex which is formed when cobra venom factor interacts with factor B, factor D and $Mg^{++}$. In both cases the reaction mixtures were passed through Sephadex G100 columns. The fractions which mediated contraction of isolated guinea-pig terminal ileum were pooled and concentrated. Cobra venom factor (CVF) was purified by the method of Bitter-Suermann *et al.* (1972). In most of the present experiments, C3a prepared with trypsin was used, but very similar results were obtained with C3a prepared by the C3 cleavage complex CVF-B-D-$Mg^{++}$.

*Target cells.*—Target cells were: P-815 cells, a chemically induced mastocytoma in a DBA/2 mouse (Dunn and Potter, 1957); L-cells, a mouse fibroblast-like cell line; D-55 cells, a derivative of 3T3 Swiss mouse cells originally described by Todaro and Green (1963); the C-243 line, originating from an agar colony of D55 cells transformed by murine sarcoma virus (MSV) (Bassin, Tuttle and Fischinger, 1970); lymph node cells obtained from mesenteric lymph nodes of BALB/c mice; Chang cells, a human liver epithelial-like cell line (Chang, 1954); and CLA-4 cells, a human B-lymphoid cell line (Steel, 1972). Human peripheral blood lymphocytes were from buffy coat residues left after platelet removal from normal human blood. Lymphocytes were separated by Ficoll-Triosil density-gradient centrifugation (Böyum, 1968). They were cultured in RPMI 1640 medium with 10% foetal bovine serum, at a concentration of $7{\cdot}5 \times 10^5$/ml and stimulated with 1 μg/ml of purified phytohaemagglutinin (Wellcome) (PHA) for 4 days. The above cell lines were maintained in the same medium.

*Labelling of cells.*—The lysis of the cells was assessed by the release of radioactive chromium from the labelled cells by the method of Brunner *et al.* (1970) and occasionally compared with the trypan blue exclusion test. For labelling of cells which grew in suspension cultures, $5 \times 10^6$ cells suspended in 1 ml of RPMI 1640 medium were incubated with 0·1 mCi [$^{51}$Cr]-sodium chromate, alone or together with 0·1 mCi [$^{86}$Rb]-rubidium chloride (The Radiochemical Centre, Amersham) for 1 h at 37 °C. The cells were washed × 4 and resuspended at a concentration of $3{\cdot}5 \times 10^5$/ml of RPMI medium containing 0·1% crystallized bovine serum albumin (BSA) (Sigma). Purified BSA was used instead of the whole serum, because of presence in the latter of an inactivator of C3a, a carboxypeptidase (Bokisch and Müller-Eberhard, 1970). In some experiments, BSA was omitted from the medium, but the tubes used in the cytotoxicity tests were rinsed with 0·1% BSA and then × 3 with phosphate-buffered saline (PBS) and spun to remove the liquid. Such treatment reduced an excessive spontaneous ıelease of the labels from the cells, which occurred in the absence of protein.

Monolayers of L-cells, D-55, C-243 and Chang cells were labelled with the same concentration of sodium chromate in tissue culture flasks, rinsed × 4 with PBS without Ca and Mg and then treated with 0·02% EDTA in PBS, pH 7·4, at 37 °C for 5–10 min to detach the cells, which were washed and suspended in the RPMI medium; clumped cells were removed by sedimentation.

*Cytotoxicity assay.*—In the cytotoxicity tests $3{\cdot}5 \times 10^4$ cells in 0·1 ml medium were mixed with various concentrations of C3 products in 0·02 ml medium in round-bottom plastic tubes (7·5 × 1 cm) and the tubes gassed with air containing 5% $CO_2$. After incubation at 37 °C for the indicated times, 1 ml of Eagle's basal medium with 5% foetal bovine serum was added to the tubes, the cells centrifuged, and 0·5-ml samples of the supernatants taken for counting of radioactivity. The percent specific release of the labels from the cells produced by C3 derivatives, was calculated as follows:

$$\frac{\text{Label released in the presence of the agent} - \text{label released in its absence}}{\text{Total label incorporated} - \text{label released in the absence of the agent}} \times 100$$

The percentage of the labels released by freezing and thawing the cells twice is indicated in the figures. Where stated, the amounts of the labels released by freezing were used, instead of the total incorporated, for the calculation of percent release. The values presented are means of duplicates and the range of variation is indicated. Spontaneous Cr release in the absence of added agents ranged between 6 and 26%, depending on the target cell type.

### RESULTS

### *Cytolytic effect of C3a on various cell types*

In several independent experiments, various types of cell were tested for their susceptibility to C3a, prepared by trypsin cleavage of the complement component C3. Fig. 1 shows that all of the 5 cell types of mouse origin tested were lysed completely or nearly completely by C3a in 6 h. Mastocytoma cells appeared to be

FIG. 1.—Lysis of various mouse cell types by C3a in 6 h. ▲—mastocytoma cells; □—lymph node cells, ■—L-cells, ○—C-243 cells, ●—D-55 cells. % label released by freezing and thawing the cells is indicated by the symbols on the ordinate.

the most sensitive, 1·2 μg/ml of C3a producing 50% lysis and 3·7 μg/ml lysis comparable to that observed after freezing and thawing. With higher doses of C3a the Cr release exceeds that produced by freezing and thawing. This was the case also with the lymph node cells. D-55 cells and their MSV-transformed derivative, C-243 cells, were lysed to a comparable extent, whereas L-cells were the least susceptible, not reaching the frozen control level even with 100 μg of C3a/ml. In another experiment, the effect of C3a on mastocytoma cells was assessed by comparing the Cr release with trypan blue exclusion of the surviving cells (Table I). There was a satisfactory agreement in values obtained by the two tests, provided that the percent Cr release was calculated by comparison with frozen cells.

In Fig. 2, the data on the lysis of 4 cell types of human origin are presented. C3a lysed CLA-4 cells and Chang cells to the same extent as lymphocytes stimulated with PHA, which were also completely lysed, whereas non-stimulated lymphocytes proved to be much more resistant.

*Effects of C3 and two of its cleavage products, C3b and C3a*

In further experiments, purified native C3, as well as its cleavage products C3a and C3b, were tested on mastocytoma cells. C3a was prepared by two different methods, employing trypsin or cobra venom factor, respectively. Cobra venom factor itself was also included as a control. As shown in Fig. 3, only C3a was found to be cytolytic, and the products prepared by the two methods did not differ appreciably in their activity. An indication of

TABLE I.—*Comparison between the Release of Chromium and Trypan Blue Exclusion by the Mastocytoma Cells Treated with C3a*

| C3a concentration μg/ml | % $^{51}$Cr release based on: Total incorporated | Releasable by freezing | 100 − % cells excluding trypan blue |
|---|---|---|---|
| 20 | 77·7±0·4 | 98·6±0·5 | 98·0±1·0 |
| 10 | 52·2±1·5 | 66·2±2·0 | 58·8±3·3 |
| 5 | 8·6±0·3 | 10·9±0·3 | 15·3±6·7 |

Incubation time, 1 h. Concentration of trypan blue, 0·2%.

FIG. 2.—Lysis of human cells by C3a in 6 h. ○—unstimulated lymphocytes, ●—PHA-stimulated lymphocytes, □—CLA-4 cells, △—Chang cells. Symbols on the ordinate indicate the freezing and thawing controls.

the potency of C3a as a tumorolytic agent is given by comparison with lysolecithin (Sigma), the latter being less active even on a weight basis. Expressed on a molar basis, C3a is much more active.

*Time course of Rb and Cr release from cells by C3a*

In several experiments, labelled Rb, a potassium analogue, was used to determine whether a small-ion marker would be released from the injured cells more rapidly than a larger marker such as Cr. This would indicate that the lysis is due to osmotic effects, as appears to be the case with complement lysis and T-lymphocyte-mediated lysis (Burakoff, Martz and Benacerraf, 1975; Ferluga and Allison, 1974). The time dependence of the release of $^{86}$Rb and $^{51}$Cr in mastocytoma cells incubated with C3a is illustrated in Fig. 4. Specific release of Rb as well as Cr reached about 40% in 7·5 min and was completed in 30 min, with 20 μg/ml of

FIG. 3.—The effect of C3, its cleavage products and other agents on mastocytoma cells. ○—C3a prepared with cobra venom factor, ●—C3a prepared with trypsin, △—C3b, ▲—cobra venom factor, □—lysolecithin, ■—C3. Time of incubation, 6 h.

C3a. A lower concentration (13 μg/ml) produced more gradual release of the markers. The specific release of Rb preceded that of Cr by about 5 min. Such a relatively short time interval between the release of a small and a large marker would indicate that the cells, after being lethally hit, undergo lysis very rapidly. Very similar results were obtained with CLA-4 cells.

*Temperature dependence of cell lysis by C3a*

The effects of temperature on the rate of release of $^{86}$Rb and $^{51}$Cr from cells in the presence of C3a are shown in Fig. 5. Mastocytoma cells incubated with 20 μg/ml C3a at 37°C for 1 h, showed nearly complete release of both markers. The release of both markers was much less in cells kept at 9°C for 1 h. However, when the cells were incubated with C3a at 37°C for 7·5 min, and then kept at 9°C for the remainder of the hour, a high proportion of both labels was still released. These results suggest that, in cells maintained at relatively low temperature, C3a is unable to exert its lytic effect. Nevertheless, brief exposure of the cells to C3a at higher temperature increases the permeability of the membrane in such a way that a subsequent fall in temperature cannot reverse it. The effect of temperature on

FIG. 4.—Time course of rubidium (Rb) and chromium (Cr) release from mastocytoma and CLA-4 cells by C3a. (A)—mastocytoma cells: solid lines, 20 μg C3a/ml, broken lines, 13·3 μg/ml. (B)—CLA-4 cells: 40 μg C3a/ml. ●, %Rb: ○, %Cr.

FIG. 5.—The influence of temperature on the release of the markers from the cells by C3a. % specific release of Rb (●) and Cr (○) from A, mastocytoma cells with 20 μg C3a/ml and B, CLA-4 cells with 40 μg C3a/ml. Solid lines, the test tubes kept at 37°C; broken lines, at 9°C for the times indicated.

the lysis of CLA-4 cells was similar to that observed with mastocytoma cells, although the differences were less pronounced. There was some lysis of CLA-4 cells maintained with C3a at 9°C, although this was much less than at 37°C. When the cells were incubated at 37°C for 10 min and transferred to 9°C, the lysis was almost stopped at the level reached in 10 min.

*Effect of serum and serum-albumin on the cell lysis by C3a*

Because serum is reported to contain an inactivator of C3a, carboxypeptidase B (Bokisch and Müller-Eberhard, 1970), guinea-pig serum, from which our C3a was derived, was tested for its effect on lysis mediated by C3a. In the presence of fresh guinea-pig serum, the lytic effect of 2·8 μg C3a was reduced to about a quarter of that observed in the absence of serum, and there was nearly a complete inhibition with lower doses of C3a (Table II). If an enzyme were involved in inactivation of C3a, it would be expected that incubation of C3a with serum for 3 h at 37°C would result in further reduction of its cytolytic capacity. However, such preincubation in 50% serum resulted in *less* inhibition of cytolysis. Similar results were obtained in other experiments. They

TABLE II.—*The Effect of Serum and Serum-albumin (BSA) on the Lysis of Mastocytoma Cells by C3a, Measured as % Specific* $^{51}$*Cr Release*

| C3a concentration (μg/ml) | Incubation in presence of | | | | | |
|---|---|---|---|---|---|---|
| | Nil (control) | | Guinea-pig serum (14% final) | | BSA 0·1% | BSA 1·0% |
| | 0* | 3* | 0 | 3 | 0 | 0 |
| 20 | 70·1±0·4 | 69·5±0·5 | 18·4±0 (73·8)† | 33·7±2·5 (51·5) | 72·9±1·8 | 71·7±1·2 |
| 10 | 68·5±0·8 | 66·9±2·7 | 2·8±0·1 (95·9) | 8·3±0·6 (87·6) | 69·9±2·0 | 63·6±1·5 |
| 5 | 5·2±2·0 | 6·1±1·4 | 0·5±0·1 (90·4) | 2·7±0·1 (55·7) | 14·2±0·5 | 16·8±0·4 |
| 0 | 17·3±1·3 | | 13·2±0·2 | | 14·2±0·2 | 13·1±0·2 |

2·8, 1·4 or 0·7 μg of C3a in 20 μl RPMI medium were mixed with 20 μl of fresh guinea-pig serum, to which 100 μl of $^{51}$Cr-labelled mastocytoma cell suspension was added, either immediately or after preincubating the C3a–serum mixtures at 37°C for 3 h, and the samples incubated for 1 h in the cytotoxicity test. Tubes for control and serum samples were rinsed with 0·1% BSA.

* Preincubation time (h).

† Numbers in parentheses, % inhibition by serum.

Label released by freezing and thawing, 72·2±1·3%.

suggest that, if C3a is modified by the action of serum enzymes (*e.g.* carboxypeptidase B), it is converted to a derivative or derivatives which are still cytolytic. In any case, the presence of even high concentrations of serum does not abolish the capacity of C3a to lyse tumour cells.

### DISCUSSION

These results establish that the complement cleavage product C3a is lytic for all the cell types tested, including several transformed cells of human and mouse origin. Our previous work (Schorlemmer and Allison, 1976) established that incubation with C3a kills mouse and guinea-pig macrophages, so the cytolytic effect may be exerted against a wide range of cell types. The lysis by C3a is highly efficient, concentrations of 1 to 10 μg/ml killing the majority of exposed cells. All the cells tested were sensitive to some degree, although normal human lymphocytes were relatively resistant. Macrophages show intermediate sensitivity to lysis by C3a. Hence, if small amounts of C3a were generated (1–10 μg/ml) tumour cells would be lysed, while macrophages and lymphocytes would be spared. Such differential effects could be important in immunity against tumours.

The concentration of C3a required for cytolysis is somewhat less than that reported to be required for mast cell degranulation (calculated from data of Cochrane and Müller-Eberhard, 1968). C3a-mediated mast cell degranulation occurs *in vivo* (Lepow *et al.*, 1970), so it is reasonable to suppose that the concentrations of C3a generated *in vivo* could have tumorolytic effects. Indeed, C3 circulates in large quantities (1·23 mg/ml), and if all were converted to C3a, the total amount in human plasma would be of the order of 50 μg/ml (Kohler and Müller-Eberhard, 1967). It is reported that C3a is inactivated by carboxypeptidase B which is present in serum (Bokisch and Müller-Eberhard, 1970). However, once the carboxy-terminal of C3a had reacted with membrane constituents, it would presumably be inaccessible to carboxypeptidase. In our experiments, incubation of C3a with fresh serum did not abolish its capacity to lyse cells, possibly because a cleavage product of C3a is still cytolytic.

The generation of C3a could be through the classical or alternative pathways of complement activation, or through enzymatic cleavage of C3 by products of activated macrophages (Schorlemmer and Allison, 1976). Tumour cells release proteinases, including plasminogen activator (Rifkin *et al.*, 1974); the active enzyme produced, plasmin, is known to cleave C3, with liberation of C3a (Bokisch, Müller-Eberhard and Cochrane, 1969). Thus it is conceivable that C3a plays a role in immunity against tumour cells, including that mediated by macrophages.

It is also possible that C3a contributes to damage of normal cells observed in some immunopathological processes when complement is activated, including some autoimmune reactions. Animals depleted of C3 for long periods (Pryjma and Humphrey, 1975) should be useful for analysing the role of C3a in protection against tumours and in immunopathology. However, C3 is produced by mononuclear phagocytes (Lai A Fat and van Furth, 1975) and may be liberated locally, even when serum C3 is depleted.

It would be interesting to examine C5a, the classical anaphylatoxin, for its possible cytolytic effect, since it is also a basic polypeptide with very similar biological properties to that of C3a (Lieflânder *et al.*, 1972). It is possible that C3a is the same as certain soluble factors which have rapid cytolytic effects, *e.g.* cytotoxic products from activated macrophages (Sethi and Brandis, 1975; Currie and Basham, 1975). Macrophages secrete C3 and an enzyme that can cleave it (Schorlemmer and Allison, 1976) and could, independently of serum constituents, generate C3a when appropriately stimulated.

The mechanism by which the lysis is brought about is not yet known. The well known effects of C3a on mast cell degranulation and contraction of smooth muscle are presumably due to increased permeability of plasma membranes to ions, especially $Ca^{++}$ (ter Laan *et al.*, 1974). The structure of C3a is known (Hugli, 1975): the polypeptide chain has an unusually cationic C-terminal region. The structure is that of an amphipathic molecule, which could become partially inserted into a membrane, with its C-terminal region interacting ionically with acidic glycoproteins and possibly glycolipids. Our observations show that incubation of cells with C3a makes their plasma membranes more permeable to ions such as $Rb^+$, an analogue of $K^+$ using the same active transport system. If there is also increased influx of $Na^+$ in excess of the active transport system, osmotic lysis would result. The fact that $^{86}Rb^+$ is released from cells before $^{51}Cr$, even by a relatively short interval, is consistent with osmotic lysis.

When cells are maintained in the presence of C3a at 9°C, there is little lysis, perhaps because C3a is unable to penetrate the membrane, which is rather rigid at that temperature; it is known that at 37–40°C membrane lipids are predominantly in a liquid phase, whereas at 9–10°C they are more closely packed in a regular hexagonal array (Engelman, 1970). When mastocytoma cells have been incubated in the presence of C3a for a few minutes at 37°C and then transferred to 9°C, loss of $^{86}Rb$ and $^{51}Cr$ continues, suggesting that once C3a is inserted on to the membrane, the increased ion flux cannot be reversed at low temperature. In contrast, the increased ion permeability produced in mastocytoma cells by T-lymphocytes was reversed at 10°C (Ferluga and Allison, 1974). Hence the two membrane lesions appear to be different.

The complement components were kindly provided by Dr D. Bitter-Suermann, Institute of Medical Microbiology, University of Mainz, Germany. D-55 and C-243 cells were obtained by courtesy of Dr R. H. Bassin. This work was supported by a grant (Scho 215/1) of the Deutsche Forschungsgemeinschaft, Bad Godesberg, Germany. L. C. Baptista and J. Ferluga are supported by the Cancer Research Campaign.

### REFERENCES

Bassin, R. H., Tuttle, N. & Fischinger, P. J. (1970) Isolation of Murine Sarcoma Virus-transformed Cells which are Negative for Leukemia Virus from Agar Suspension Cultures. *Int. J. Cancer*, **6**, 95.

Bitter-Suermann, D., Dierich, M., König, W. & Hadding, U. (1972) Bypass-activation of the Complement System Starting with C3. I. Generation and Function of an Enzyme from a Factor of Guinea-pig Serum and Cobra Venom. *Immunology*, **23**, 267.

Bitter-Suermann, D., Hadding, U., Melchert, F. & Wellensiek, H. J. (1970) Independent and Consecutive Action of C5, C6 and C7 in Immune Hemolysis. I. Preparation of EAC1–5 with Purified Guinea Pig C3 and C5. *Immunochemistry*, **7**, 955.

BOKISCH, V. A. & MÜLLER-EBERHARD, H. J. (1970) Anaphylatoxin Inactivator of Human Plasma: its Isolation and Characterization as a Carboxypeptidase. *J. clin. Invest.*, **49**, 2427.

BOKISCH, V. A., MÜLLER-EBERHARD, H. J. & COCHRANE, C. G. (1969) Isolation of a Fragment (C3a) of the Third Component of Human Complement Containing Anaphylatoxin and Chemotactic Activity and Description of an Anaphylatoxin Inactivator of Human Serum. *J. exp. Med.*, **129**, 1109.

BÖYUM, A. (1968) Separation of Leukocytes from Blood and Bone Marrow. *Scan. J. clin. Lab. Invest.*, **21** (Suppl. 97), 51.

BRUNNER, K. T.. MAUEL, J., RUDOLF, H. & CHAPUIS, B. (1970) Studies on Allograft Immunity in Mice. I. Induction, Development and *in vitro* Assay of Cellular Immunity. *Immunology*, **18**, 499.

BURAKOFF, S. J., MARTZ, E. & BENACERRAF, B. (1975) Is the Primary Complement Lesion Insufficient for the Lysis? Failure of Cells Damaged under Osmotic Protection to Lyse in EDTA or at Low Temperature after Removal of Osmotic Protection. *Clin. Immunol. Immunopath.*, **4**, 108.

CEROTTINI, J. C. & BRUNNER, K. T. (1974) Cell-mediated Cytotoxicity, Allograft Rejection and Tumour Immunity. *Adv. Immunol.*, **18**, 67.

CHANG, R. S. (1954) Continuous Subcultivation of Epithelial-like Cells from Normal Human Tissues. *Proc. Soc. exp. Biol. Med.*, **87**, 440.

COCHRANE, C. G. & MÜLLER-EBERHARD, H. J. (1968) The Derivation of Two Distinct Anaphylatoxin Activites from the Third and Fifth Components of Human Complement. *J. exp. Med.*, **127**, 371.

CURRIE, G. A. & BASHAM, C. (1975) Activated Macrophages Release a Factor Which Lyses Malignant Cells but not Normal Cells. *J. exp. Med.*, **142**, 1600.

DIAS DA SILVA, W., EISELE, J. W. & LEPOW, I. H. (1967) Complement as a Mediator of Inflammation. III. Purification of the Activity with Anaphylatoxin Properties Generated by Interaction of the First Four Components of Complement and its Identification as a Cleavage Product of C′3. *J. exp. Med.*, **126**, 1027.

DUNN, T. B. & POTTER, M. (1957) A Transplantable Mast-cell Neoplasm in the Mouse. *J. natn. Cancer Inst.*, **18**, 587.

EIFEL, P. J., WALKER, S. M. & LUCAS, Z. J. (1975) Standardization of a Sensitive and Rapid Assay for Lymphotoxin. *Cell. Immunol.*, **15**, 208.

ENGELMAN, D. M. (1970) X-ray Diffraction Studies of Phase Transitions in the Membrane of *Mycoplasma laidlawii*. *J. mol. Biol.*, **47**, 115.

EVANS, R. & ALEXANDER, P. (1971) Rendering Macrophages Specifically Cytotoxic by a Factor Released from Immune Lymphoid Cells. *Transplantation*, **12**, 227.

FERLUGA, J. & ALLISON, A. C. (1974) Observations on the Mechanism by which T-lymphocytes exert Cytotoxic Effects. *Nature, Lond.*, **250**, 673.

HUGLI, T. E. (1975) Human Anaphylatoxin (C3a) from the Third Component of Complement. Primary Structure. *J. biol. Chem.*, **250**, 8293.

KOHLER, P. F. & MÜLLER-EBERHARD, H. J. (1967) Immunochemical Quantitation of the Third, Fourth and Fifth Components of Human Complement. Concentrations in the Serum of Healthy Adults. *J. Immunol.*, **99**, 1211.

TER LAAN. B., MOLENAAR, J. L., FELDKAMP-VROOM, T. M. & PONDMAN, K. W. (1974) Interaction of Human Anaphylatoxin C3a with Rat Mast Cells Demonstrated by Immunofluorescence. *Eur. J. Immunol.*, **4**, 393.

LAI A. FAT, R. M. & VAN FURTH, R. (1975) *In vitro* Synthesis of Some Complement Components (Clq, C3 and C4) by Lymphoid Tissues and Circulating Leukocytes in Man. *Immunology*, **28**, 359.

LEPOW, I. H., WILLMS-KRETSCHMER, K., PATRICK, R. A. & ROSEN, F. S. (1970) Gross and Ultrastructural Observations on Lesions Produced by Intradermal Injection of Human C3a in Man. *Am. J. Pathol.*, **61**, 13.

LIEFLÄNDER, M., DIELENBERG, D., SCHMIDT, O. & VOGT, W. (1972) Structural Elements of Anaphylatoxin Obtained by Contact Activation of Hog Serum. *Hoppe-Seyler's Z. Physiol. Chem.*, **353**, 385.

NICHOLSON, A., BRADE, V., SCHORLEMMER, H. U., BURGER, R., BITTER-SUERMANN, D. & HADDING, U. (1975) Interaction of C3b, B and D in the Alternative Pathway of Complement Activation. *J. Immunol.*, **115**, 1108.

OLD, L. J., STOCKERT, E., BOYSE, E. A. & GEERING, G. (1967) A Study of Passive Immunization against a Transplanted G + Leukemia with Specific Antiserum. *Proc. Soc. exp. Biol. Med.*, **124**, 63.

PERLMANN, P. & HOLM, G. (1969) Cytotoxic Effects of Lymphoid Cells *in vitro*. *Advan. Immunol.*, **11**, 117.

PRYJMA, J. & HUMPHREY, J. H. (1975) Prolonged C3 Depletion by Cobra Venom Factor in Thymus-deprived Mice and its Implication for the Role of C3 as an Essential Second Signal for B-cell Triggering. *Immunology*, **28**, 569.

RIFKIN, D. B., LEOB, J. N., MOORE, G. & REICH, E. (1974) Properties of Plasminogen Activators Formed by Neoplastic Human Cell Cultures. *J. exp. Med.*, **139**, 1317.

SCHORLEMMER, H. U. & ALLISON, A. C. (1976) Effects of Activated Complement Components on Enzyme Secretion by Macrophages. *Immunology*, in press.

SCHORLEMMER, H. U., DAVIES, P. & ALLISON, A. C. (1976) Ability of Activated Complement Components to Induce Lysosomal Enzyme Release from Macrophages. *Nature, Lond.*, **261**, 48.

SETHI, K. K. & BRANDIS, H. (1975) Cytotoxicity Mediated by Soluble Macrophage Product(s). *J. natn. Cancer Inst.*, **55**, 393.

STEEL, C. M. (1972) Human Lymphoblastoid Cell Lines. III. Co-cultivation Technique for Establishment of New Lines. *J. natn. Cancer Inst.*, **48**, 623.

TODARO, G. J. & GREEN, H. (1963) Quantitative Studies of the Growth of Mouse Embryo Cells in Culture and their Development into Established Lines. *J. Cell Biol.*, **17**, 299.